# Oral and Dental Findings of Dyskeratosis Congenita

**DOI:** 10.1155/2014/454128

**Published:** 2014-12-24

**Authors:** Mine Koruyucu, Pelin Barlak, Figen Seymen

**Affiliations:** Faculty of Dentistry, Department of Pedodontics, Istanbul University, 34093 Istanbul, Turkey

## Abstract

Dyskeratosis congenital (DC) is a rare condition characterized by reticulate skin hyperpigmentation, mucosal leukoplakia, and nail dystrophy. More serious features are bone marrow involvement with pancytopenia and a predisposition to malignancy. The purpose of this case report is to describe the oral and dental findings in children with DC syndrome. A 10-year-old male diagnosed with DC was admitted because of extensive caries and toothache. Inadequate oral hygiene and extensive caries were observed in oral examination of the patient. Plaque accumulation was seen in gingival border of maxillary teeth. Papillary atrophy on the tongue was observed. Short and blunted roots of mandible incisors and upper and lower molars were determined on the radiographic examination. Dryness on the lips and commisuras, ectropion on his eyes, and epiphora were observed. Hematologic tests were performed and showed aplastic anemia at the age of 2. At the age of 4, the bone marrow transplantation was performed. Dermatological findings occurred after the bone marrow transplantation. The skin of the patient was thin, dry, and wrinkled in some areas. He had palmoplantar hyperkeratosis and syndactylia on his fingers. Endodontic treatment procedures were applied and other extensive caries are still being restored. The patient will be given full preventive care during regular follow-up. Oral hygiene was improved to the optimum level.

## 1. Introduction

Dyskeratosis congenita (DC) is a rare inherited disorder with a prevalence of less than one per million. The mode of inheritance is uncertain. The most common mode of inheritance is the X-linked recessive form which affects mainly males and is caused by mutation of the DKC1 gene at the Xq28 site [1–4].

In the early 1900s, Zinsser et al. described an inherited variant of ectodermal dysplasia that affected skin, nails, and mucous membranes [4–7]. The syndrome eventually became known as DC and is classified as one of the inherited bone marrow failure syndromes (IBMFS) [7]. DC was defined by the association of three clinical features: dystrophic nails, oral leukoplakia (white spots on the tongue and oral mucosa), and abnormal skin pigmentation [8]. Although the principal clinical findings of these conditions are described as a triad, there is a high association of hematopoietic abnormalities with this syndrome [9–12]. The relevance of this case of DC was the unusual presentation and progression of the clinical features without the so-called “classic triad” of features with which the disease normally presents. The condition usually follows a classic pattern: nail dystrophy develops at 6, oral white plaques by 7, and abnormal reticulate skin pigmentation at 8 years of age. The earliest sign may be either anemia or thrombocytopenia, but ultimate progression to pancytopenia is usual [1]. Multisystem clinical features of DC clinical feature/abnormality were observed in Table 1 [13].

DC usually starts between the ages of 5 and 12 with dermatologic symptoms. Pigmentation of the skin and dystrophy and atrophy of the nails are the most frequent manifestations [9, 11, 12, 14]. Diagnosis may be delayed until clinical signs are apparent. Severe pancytopenia frequently causes early mortality of DC patients [6]. Patients with DC have a predisposition for the development of a variety of malignancies, in particular myelodysplastic syndrome (MDS) and secondary acute myeloid leukemia (AML), head/neck cancer, and esophageal cancer [15, 16]. Malignancies tend to occur earlier in life compared to the same malignancies in non-DC individuals and are often the cause of death of patients in the third, fourth, and fifth decade of life. Individuals with DC may develop independent tumors at more than one site [2, 8, 15].

Oral and dental abnormalities have been reported in a few cases and include hypodontia, short blunted roots, hypocalcification, thin enamel, gingival recession, gingival inflammation with oedema, gingival bleeding, alveolar bone loss, periodontitis, extensive caries, smooth atrophic tongue mucosa, leukoplakia, and lichen planus [4] (Table 2). 

## 2. Case Report

A 10-year-old male diagnosed with dyskeratosis congenita was admitted because of extensive caries and toothache to Department of Pediatric Dentistry of Istanbul University.

**Figure 1:**
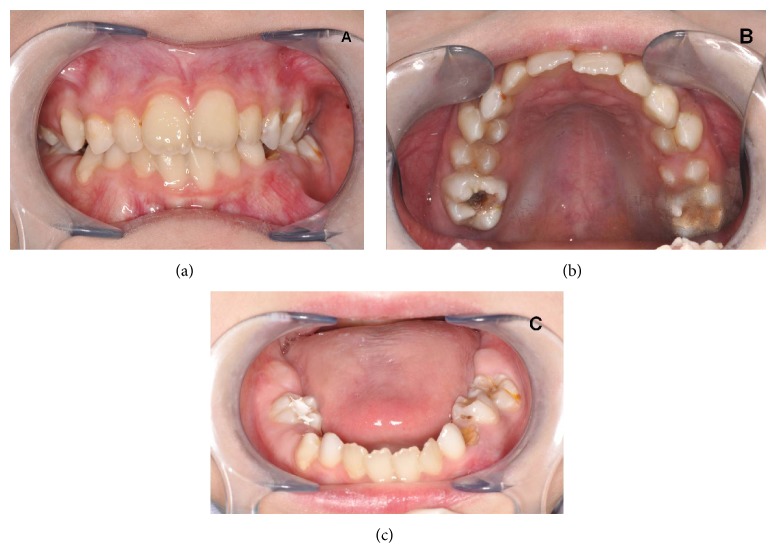
((a), (b), and (c)) Intraoral view.

Inadequate oral hygiene (DMFT = 7) and extensive caries were observed in the oral examination of the patient. The mandibular and maxillary first permanent molars and primary teeth (53, 74, 75) had carious lesions; also white spot lesions were seen in teeth 63 and 14. Plaque accumulation was seen in gingival border of maxillary teeth. The tongue was normal in size but papillary atrophy on the tongue was observed (Figures 1(a), 1(b), and 1(c)).

**Figure 2:**
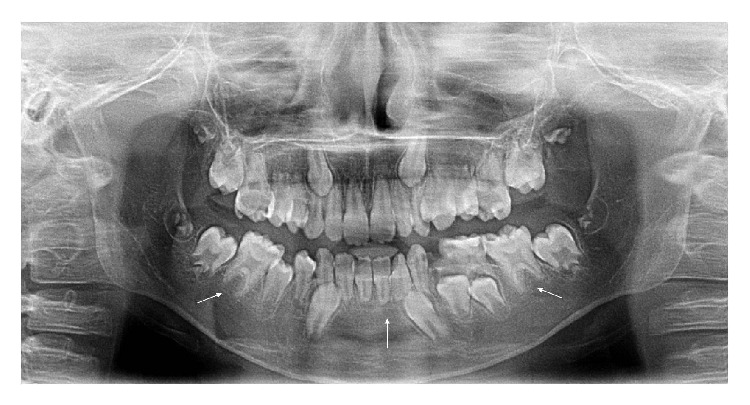
Panoramic view.

Short and blunted roots of mandible incisors and upper and lower molars were determined on the radiographic examination. Decreased root/crown ratios were found in multiple teeth (Figure 2).

**Figure 3:**
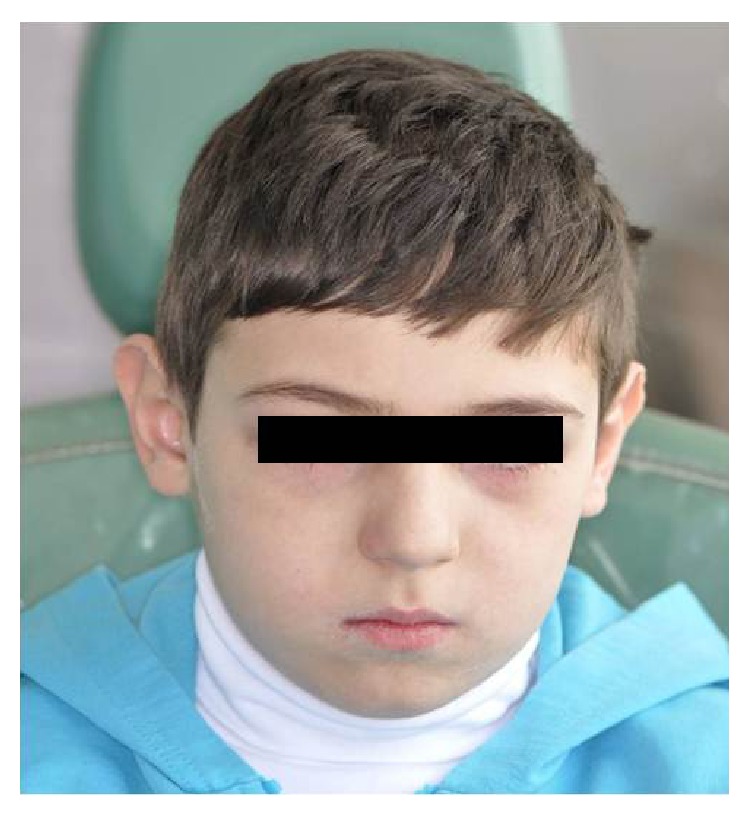
Extraoral view.

His commisuras were dry and irritated (Figure 3); patient is forced when opening his mouth widely.

Patient appeared pale but vital signs were within normal limits. Dryness on the lips, ectropion on his eyes, and epiphora were observed (Figure 3). Epiphora occurs as a result of hyperplasia of the epithelial lining of the lacrimal punctum.

**Figure 4:**
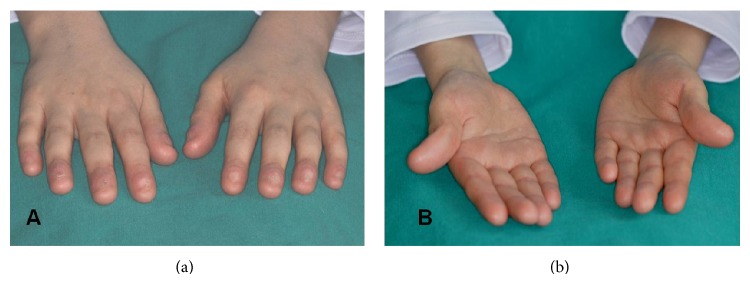
Hand view.

The first dermatological complaint of the patient was extensive petechiae on his skin when he was 2. Hematologic tests were performed and the result showed aplastic anemia. There was no parental consanguinity and no other relevant family history; his sister was not affected. At the age of 4, the bone marrow transplantation was performed. Dermatological findings occurred after the bone marrow transplantation. The skin of the patient was thin, dry, and wrinkled in some areas. He had palmoplantar hyperkeratosis and syndactylia on his fingers. His fingers had rudimentary nails. The nails were totally lost on some fingers (Figures 4(a) and 4(b)).

The patient was suffering from toothache. Endodontic treatment procedures were applied (primary molar teeth) and other extensive caries are still being restored (permanent teeth). The patient will be given full preventive care during regular follow-up. Oral hygiene was improved to the optimum level. Orthodontic consultation was taken for his mild malocclusion.

## 3. Discussion

Dyskeratosis congenita is a very rare syndrome. DC usually starts between the ages of 5 and 12 and it has oral findings [5, 10, 16, 18]. In this case, DC was identified 5 years earlier. Of the published cases, most of them are males. The reported male to female ratio is 13 : 1 [2]. Our case is also male.

Several case reports have described oral changes in DC, which include destruction of alveolar margins, gingival inflammation, hypomineralized teeth, oral leukoplakia, increased dental caries, hypodontia, thin enamel structure, aggressive periodontitis, intraoral brown pigmentation, tooth loss, taurodontism, and short blunted roots [5, 10, 16, 18].

Brown were indicated that white patches of necrotic epithelium or possible candida infection in age of 5–14. Recurrent ulceration and erythroplakia at the age of 14–20 and erosive leukoplakia and carcinoma in age of 20–30 [2].

Early childhood caries is a more common feature, affecting 17% of DC patients, together with other dental abnormalities, including short blunted roots and periodontal disease [5–7]. These changes are thought to be due to anomalies within structures of ectodermal origin, which result in defects of the enamel organ or its epithelial attachment [1]. In this case, short blunted roots, extensive carious lesions, and minimal plaque accumulation were determined and, according to parent's anamnesis, he also had early childhood caries.

Atkinson et al. examined 17 individuals with DC and the most commonly found oral changes were oral leukoplakia (65%), decreased root/crown ratio (75%), and mild taurodontism (57%) [5]. Leukoplakia of the tongue developed in Brown's case at the age of seven [2]. Although oral leukoplakia is one of the major oral findings in patients (87%) who suffer from DC, there was no leukoplakia in our patient's oral cavity. Decreased root/crown ratio and mild taurodontism were determined in the radiography. Since root length is reduced in the DC patients, it was not surprising that taurodontism was found in our case.

Root development begins after the crown of the tooth is formed. The earliest root development of the permanent teeth occurs at about the age of 3 years, beginning with the central incisors [5, 8, 17, 19]. The second permanent molar roots begin to form between 7 and 8. Insults to the developing teeth, such as radiation, chemotherapy, or hematopoietic stem cell transplant during that time, can disturb root development [8].

In Atkinson et al.'s examination, intraoral soft tissue changes were found in 12 of 17 DC patients. Their findings were classified by appearance and by location into the following categories: leukoplakia (excluding prominent linea alba), erythema, brown pigmentation, and papillary atrophy on the tongue [5]. In our case, we determined papillary atrophy on the tongue.

Davidovich et al. examined a case with DC; radiographs revealed extensive alveolar bone loss in all quadrants extending from the primary canines to the second primary molars [3]. In this case, we did not observe alveolar bone loss.

Also Davidovich et al. summarize the preoperative, operative, and postoperative considerations that are important in such cases as follows.

*Dental Management of a Child with Dyskeratosis Congenita Undergoing Bone Marrow Transplantation [3]*.

- * Preoperative considerations.*
- * Consultation.*
   Confirm diagnosis and status of medical condition.
   Consult hematologist for blood parameters and pulmonary infections as well as medications.
   Consult anesthesiologist for possible risk of respiratory complications under general anesthesia (esophageal webbing or stenosis and bronchitis).
   Consult a periodontist for periodontal status (gingival inflammation, recessions, and alveolar bone loss).
- * Goals of dental treatment.*
   Being free from acute infection.
   Being free from potential dental infections: eliminate decay and restore.
   Being free from potential gingival infection: eliminate inflammation.
   Observing the oral mucosa for ulcers and leukoplakia.
   Contributing to meticulous oral hygiene and prevention protocol (chlorhexidine, fluoride, and cleaning).
- * Operative considerations.*
   Perform meticulous hematologic preparation before and during the treatment under general anesthesia.
   Confirm status of RBC, WBC, and platelets.
   Administer platelets from single platelets donor (SPD) if necessary.
   Administer tranexamic acid (Hexakapron).
   Administer amoxicillin IV as prophylaxis for infection during the dental treatment.
   Control bleeding by local means.
   Administer balanced anesthesia.
   Meticulously eliminate plaque and apply fluoride (gel or varnish).
   Radical dental treatment approach is recommended.
   Restorations: use amalgam or glass ionomer when potential wet area is suspected.
   Avoid stainless steel crowns.
   Extract mobile teeth and teeth with questionable prognosis.
- * Postoperative considerations.*
   Monitor the patient in PACU for a longer period of time than is required for a regular dental care.
   Administer tranexamic acid for the next 48 hours after dental treatment to ensure adequate hemostasis.
   Administer antibiotics for 48 hours after dental treatment to ensure infection control due to anesthesia and dental treatment.

The dental treatment must follow the treatment goals. Restorative materials may include amalgam or glass ionomer when a dry field cannot be achieved. It is important to apply preventive means such as fluoride varnishes [3]. Our patient will be given full preventive care after restorative treatment.

## 4. Conclusion

These patients need the multidisciplinary approach in the dental and systemic treatment of a child suffering from dyskeratosis congenita. The roles of the hematologist, anesthesiologist, periodontist, and pediatric dentist are crucial in the planning and performing of the dental treatment. Regular follow-up of the patient is essential owing to the possibility of malignant changes within oral and other mucosal sites.

## Figures and Tables

**Table 1:** Multisystem clinical features of DC clinical feature/abnormality [4, 13].

	% of patients
Major/common features	
Abnormal skin pigmentation	89
Nail dystrophy	88
BM failure	85.5
Leukoplakia	78
Other recognized somatic features	
Epiphora	30.5
Learning difficulties/developmental delay/mental retardation	25.4
Pulmonary disease	20.3
Short stature	19.5
Extensive dental caries/loss	16.9
Esophageal stricture	16.9
Premature hair loss/greying/sparse eyelashes	16.1
Hyperhidrosis	15.3
Malignancy	9.8
Intrauterine growth retardation	7.6
Liver disease/peptic ulceration/enteropathy	7.3
Ataxia/cerebellar hypoplasia	6.8
Hypogonadism/undescended testes	5.9
Microcephaly	5.9
Urethral stricture/phimosis	5.1
Osteoporosis/aseptic necrosis/scoliosis	5.1
Deafness	0.8

**Table 2:** Oral and dental findings in dyskeratosis congenita.

Findings	Literatures
Dental findings	
Hypodontia	Brown [2], Baran et al. [4], and Atkinson et al. [5].
Short blunted roots	Abdel-Karim et al. [1], Brown [2], Atkinson et al. [5], Hölttä et al. [8], and Thomas [17].
Extensive caries lesions	Abdel-Karim et al. [1], Davidovich et al. [3], and Atkinson et al. [5].
Delayed eruption	Brown [2].
Taurodontism	Baran et al. [4] and Atkinson et al. [5].
Periodontal findings	
Gingival inflammation with oedema	Brown [2] and Baran et al. [4].
Alveolar bone loss	Abdel-Karim et al. [1], Brown [2], Davidovich et al. [3], Baran et al. [4], and Atkinson et al. [5].
Periodontitis	Davidovich et al. [3] and Atkinson et al. [5].
Thin enamel gingival recession	Atkinson et al. [5].
Smooth atrophic tongue mucosa	Abdel-Karim et al. [1], Brown [2], Atkinson et al. [5], and Dokal [13].
Leukoplakia	Abdel-Karim et al. [1], Brown [2], Davidovich et al. [3], Baran et al. [4], Atkinson et al. [5], Zinsser [7], Ogden et al. [10], and Davidson and Connor [18].
Dry mouth	Baran et al. [4].
Angular cheilitis	Baran et al. [4].
